# 1-(4-Chloro­phen­yl)-3-phenyl-1*H*-pyrazol-5(4*H*)-one

**DOI:** 10.1107/S1600536809055263

**Published:** 2010-02-27

**Authors:** Yong-Jie Ding, Chun-Xiang Zhao

**Affiliations:** a Department of Chemistry, East China Normal University, Shanghai 200062, People’s Republic of China; b Department of Chemistry, Zhoukou Normal University, Zhoukou 466001, People’s Republic of China

## Abstract

In the crystal of the title compound, C_15_H_11_ClN_2_O, the molecules are linked by C—H⋯O and weak C—H⋯π inter­actions. The chloro­phenyl and phenyl rings are twisted with respect to the central pyrazolone ring, making dihedral angles of 18.23 (8) and 8.35 (8)°, respectively. The N—N and C=O bond lengths are comparable to those reported for pyrazolone compounds.

## Related literature

For the properties and applications of pyrazolones and their derivatives, see: Bao *et al.* (2006 ▶); Bose *et al.* (2005 ▶); Ito *et al.* (2001 ▶); Li *et al.* (2000 ▶); Shi, *et al.* (2005 ▶); Whitaker (1995 ▶). For the synthesis, see: Jensen (1959 ▶). For related structures, see: Bovio *et al.* (1974 ▶); Dardonville *et al.* (1998 ▶); Ferretti *et al.* (1985 ▶); Holzer *et al.* (1999 ▶).
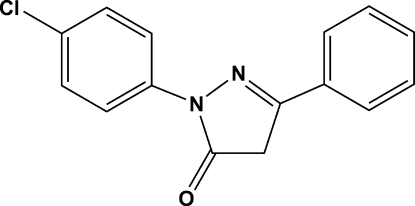


## Experimental

### 

#### Crystal data

C_15_H_11_ClN_2_O
*M* *_r_* = 270.71
Monoclinic, 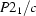

*a* = 11.2593 (4) Å
*b* = 12.1848 (4) Å
*c* = 9.5498 (3) Å
β = 103.053 (1)°
*V* = 1276.31 (7) Å^3^
*Z* = 4
Mo *K*α radiation
μ = 0.29 mm^−1^
*T* = 296 K
0.32 × 0.28 × 0.15 mm

#### Data collection

Bruker APEXII CCD area-detector diffractometer
17039 measured reflections
3172 independent reflections
2578 reflections with *I* > 2σ(*I*)
*R*_int_ = 0.021

#### Refinement

*R*[*F* ^2^ > 2σ(*F* ^2^)] = 0.039
*wR*(*F* ^2^) = 0.111
*S* = 1.04
3172 reflections
172 parameters
H-atom parameters constrained
Δρ_max_ = 0.23 e Å^−3^
Δρ_min_ = −0.20 e Å^−3^

### 

Data collection: *APEX2* (Bruker, 2005 ▶); cell refinement: *SAINT* (Bruker, 2005 ▶); data reduction: *SAINT*; program(s) used to solve structure: *SHELXS97* (Sheldrick, 2008 ▶); program(s) used to refine structure: *SHELXL97* (Sheldrick, 2008 ▶); molecular graphics: *SHELXTL* (Sheldrick, 2008 ▶); software used to prepare material for publication: *SHELXL97*.

## Supplementary Material

Crystal structure: contains datablocks I, global. DOI: 10.1107/S1600536809055263/dn2522sup1.cif

Structure factors: contains datablocks I. DOI: 10.1107/S1600536809055263/dn2522Isup2.hkl

Additional supplementary materials:  crystallographic information; 3D view; checkCIF report

## Figures and Tables

**Table 1:** Hydrogen-bond geometry (Å, °) *Cg*1 is the centroid of the C10–C15 ring.

*D*—H⋯*A*	*D*—H	H⋯*A*	*D*⋯*A*	*D*—H⋯*A*
C8—H8*B*⋯O1^i^	0.97	2.40	3.3115 (19)	156
C8—H8*A*⋯*Cg*1^ii^	0.97	2.76	3.5026 (17)	134

Symmetry codes: (i) 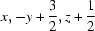; (ii) 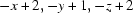.

**Table 2:** Comparison of C=O and N—N bond lengths (Å) between the title compound and reported pyrazolone compounds.

Compound	C=O	N—N
C_13_H_14_N_2_O_2_^*a*^	1.313 (2)	1.395 (2)
C_19_H_16_N_2_O_2_^*a*^	1.261 (2)	1.404 (2)
C_15_H_12_N_2_O_2_S^*a*^	1.246 (2)	1.373 (2)
C_22_H_15_ClN_2_O^*c*^	1.228 (2)	1.405 (2)
C_16_H_11_N_3_O^*c*^	1.252 (3)	1.412 (4)
C_16_H_10_ClN_3_O^*c*^	1.250 (5)	1.420 (5)
C_10_H_8_N_4_O_5_^*d*^	1.207 (3)	1.412 (2)
C_15_H_11_ClN_2_O^*e*^	1.213 (2)	1.404 (2)

Notes: (*a*) Holzer *et al.* (1999 ▶); (*b*) Bovio *et al.* (1974 ▶); (*c*) Ferretti *et al.* (1985 ▶); (*d*) Dardonville *et al.* (1998 ▶); (*e*) this work.

**Fig. 1.**
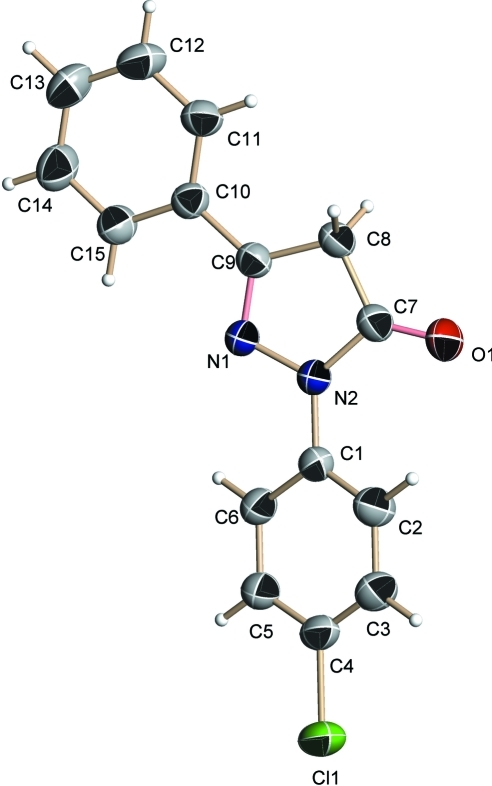
The molecular structure of (I) (thermal ellipsoids are shown at 30% probability levels).

## supplementary crystallographic information

### Comment

Pyrazolones and their derivatives constitute a group of organic compounds that have been extensively studied due to their diverse properties and applications. For example, many more applications have been devised for this group of molecules in the pharmaceutical field. Moreover, they have been applied to the solvent extraction of metal ions (Bose *et al.*, 2005), for analytical purposes (Ito *et al.*, 2001), in the preparation of azo colorants (Whitaker, 1995), as ligands in complexes with catalytic activity (Bao *et al.*, 2006) and in the synthesis of rare earth metal complexes with interesting photophysical properties (Shi *et al.*, 2005). Also, it is important in understanding the behaviour of these compounds with respect to the mechanisms of pharmacological activities (Li *et al.*, 2000). In order to expand this field, the novel title compound (I) has been synthesized, and its crystal structure is reported herein for the first time.

The asymmetric unit of the title compound is built up from a central pyrazolone ring substituted in 1,3 by a 4-chlorophenyl and a phenyl rings (Fig. 1). The chlorophenyl and phenyl rings are slightly twisted with respect to the central pyrazolone ring making dihedral angles of 18.23 (8)° and 8.35 (8)° respectively, thus indicating a high degree of conjugation and electron delocalization.The N(1)–N(2) and C(7)=O(1) distances are comparable, within experimental errors, with related pyrazolones reported in the literature (Table 2).

The cohesion of the crystal is assured by weak C-H···O and C-H···π interactions (Table 1).

### Experimental

All reagents were obtained from commercial sources and used without further purification. 1-(4-chlorophenyl)-3-phenyl-1*H*-pyrazol-5(4*H*)-one was synthesized according to the method proposed by Jensen (1959). (yield 84.5%; m.p. 435–436 K). Analysis required for C_15_H_11_ClN_2_O: C 66.55%, H 4.10%, N10.35%; found: C 66.51, H 4.08, N 10.41%. Block-like golden single crystals of CPP were grown from ethanol by slow evaporation over a period of several weeks.

### Refinement

All H atoms were placed in calculated positions, with C—H = 0.93Å for phenyl and 0.97 Å for methylene, and treated as riding with *U*_iso_(H) =1.2*U*_eq_ (C) .

### Crystal data

**Table:**

C_15_H_11_ClN_2_O	*F*(000) = 560.0
*M**_r_* = 270.71	*D*_x_ = 1.409 Mg m^−^^3^
Monoclinic, *P*2_1_/*c*	Mo *K*α radiation, λ = 0.71073 Å
Hall symbol: -P 2ybc	Cell parameters from 8730 reflections
*a* = 11.2593 (4) Å	θ = 2.5–28.4°
*b* = 12.1848 (4) Å	µ = 0.29 mm^−^^1^
*c* = 9.5498 (3) Å	*T* = 296 K
β = 103.053 (1)°	Block, yellow
*V* = 1276.31 (7) Å^3^	0.32 × 0.28 × 0.15 mm
*Z* = 4	

### Data collection

**Table:**

Bruker SMART CCD area-detector diffractometer	2578 reflections with *I* > 2σ(*I*)
Radiation source: fine-focus sealed tube	*R*_int_ = 0.021
graphite	θ_max_ = 28.4°, θ_min_ = 1.9°
φ and ω scans	*h* = −14→15
17039 measured reflections	*k* = −15→16
3172 independent reflections	*l* = −12→11

### Refinement

**Table:**

Refinement on *F*^2^	Primary atom site location: structure-invariant direct methods
Least-squares matrix: full	Secondary atom site location: difference Fourier map
*R*[*F*^2^ > 2σ(*F*^2^)] = 0.039	Hydrogen site location: inferred from neighbouring sites
*wR*(*F*^2^) = 0.111	H-atom parameters constrained
*S* = 1.04	*w* = 1/[σ^2^(*F*_o_^2^) + (0.0526*P*)^2^ + 0.3591*P*] where *P* = (*F*_o_^2^ + 2*F*_c_^2^)/3
3172 reflections	(Δ/σ)_max_ < 0.001
172 parameters	Δρ_max_ = 0.23 e Å^−^^3^
0 restraints	Δρ_min_ = −0.20 e Å^−^^3^

### Special details

**Table:**

Geometry. All e.s.d.'s (except the e.s.d. in the dihedral angle between two l.s. planes) are estimated using the full covariance matrix. The cell e.s.d.'s are taken into account individually in the estimation of e.s.d.'s in distances, angles and torsion angles; correlations between e.s.d.'s in cell parameters are only used when they are defined by crystal symmetry. An approximate (isotropic) treatment of cell e.s.d.'s is used for estimating e.s.d.'s involving l.s. planes.
Refinement. Refinement of *F*^2^ against ALL reflections. The weighted *R*-factor *wR* and goodness of fit *S* are based on *F*^2^, conventional *R*-factors *R* are based on *F*, with *F* set to zero for negative *F*^2^. The threshold expression of *F*^2^ > σ(*F*^2^) is used only for calculating *R*-factors(gt) *etc*. and is not relevant to the choice of reflections for refinement. *R*-factors based on *F*^2^ are statistically about twice as large as those based on *F*, and *R*- factors based on ALL data will be even larger.

### Fractional atomic coordinates and isotropic or equivalent isotropic displacement parameters (Å^2^)

**Table:**

	*x*	*y*	*z*	*U*_iso_*/*U*_eq_	
Cl1	0.49588 (4)	0.36865 (4)	0.10838 (4)	0.06295 (16)	
N2	0.73364 (11)	0.53865 (9)	0.68321 (12)	0.0405 (3)	
N1	0.75985 (10)	0.46368 (9)	0.79773 (12)	0.0396 (3)	
C7	0.76387 (14)	0.64516 (11)	0.72628 (15)	0.0442 (3)	
C8	0.82119 (14)	0.63688 (11)	0.88421 (15)	0.0434 (3)	
H8A	0.9060	0.6593	0.9047	0.052*	
H8B	0.7777	0.6811	0.9407	0.052*	
C1	0.67906 (12)	0.49854 (11)	0.54432 (14)	0.0380 (3)	
C4	0.56921 (13)	0.41944 (13)	0.27598 (14)	0.0445 (3)	
O1	0.74620 (13)	0.72658 (9)	0.65145 (12)	0.0620 (3)	
C5	0.56870 (15)	0.35700 (12)	0.39612 (16)	0.0491 (3)	
H5	0.5317	0.2883	0.3866	0.059*	
C10	0.85009 (12)	0.46390 (11)	1.05204 (14)	0.0392 (3)	
C9	0.80911 (12)	0.51759 (11)	0.91237 (14)	0.0375 (3)	
C3	0.62544 (16)	0.51956 (13)	0.28785 (16)	0.0538 (4)	
H3	0.6260	0.5607	0.2060	0.065*	
C15	0.82395 (14)	0.35388 (12)	1.07152 (17)	0.0476 (3)	
H15	0.7787	0.3135	0.9952	0.057*	
C6	0.62340 (13)	0.39676 (12)	0.53086 (15)	0.0448 (3)	
H6	0.6228	0.3551	0.6123	0.054*	
C11	0.91727 (14)	0.52294 (13)	1.16804 (15)	0.0481 (3)	
H11	0.9340	0.5967	1.1569	0.058*	
C13	0.93286 (17)	0.36390 (16)	1.31805 (18)	0.0617 (4)	
H13	0.9606	0.3304	1.4069	0.074*	
C2	0.68150 (16)	0.55939 (13)	0.42195 (16)	0.0531 (4)	
H2	0.7209	0.6270	0.4303	0.064*	
C12	0.95927 (16)	0.47258 (15)	1.29984 (16)	0.0582 (4)	
H12	1.0054	0.5122	1.3763	0.070*	
C14	0.86514 (16)	0.30462 (15)	1.20418 (18)	0.0594 (4)	
H14	0.8472	0.2313	1.2168	0.071*	

### Atomic displacement parameters (Å^2^)

**Table:**

	*U*^11^	*U*^22^	*U*^33^	*U*^12^	*U*^13^	*U*^23^
Cl1	0.0769 (3)	0.0676 (3)	0.0378 (2)	0.0076 (2)	−0.00071 (18)	−0.01026 (16)
N2	0.0514 (6)	0.0331 (5)	0.0348 (5)	−0.0008 (5)	0.0052 (5)	0.0022 (4)
N1	0.0474 (6)	0.0345 (6)	0.0352 (5)	−0.0001 (5)	0.0058 (5)	0.0031 (4)
C7	0.0551 (8)	0.0358 (7)	0.0416 (7)	−0.0025 (6)	0.0108 (6)	−0.0011 (5)
C8	0.0559 (8)	0.0347 (7)	0.0392 (7)	−0.0025 (6)	0.0101 (6)	−0.0032 (5)
C1	0.0415 (7)	0.0371 (7)	0.0347 (6)	0.0030 (5)	0.0071 (5)	−0.0001 (5)
C4	0.0479 (7)	0.0494 (8)	0.0342 (6)	0.0093 (6)	0.0053 (5)	−0.0045 (6)
O1	0.0983 (9)	0.0359 (5)	0.0487 (6)	−0.0051 (5)	0.0098 (6)	0.0068 (5)
C5	0.0574 (9)	0.0413 (7)	0.0435 (7)	−0.0040 (6)	0.0007 (6)	−0.0003 (6)
C10	0.0409 (7)	0.0420 (7)	0.0354 (6)	0.0008 (5)	0.0100 (5)	0.0003 (5)
C9	0.0408 (6)	0.0354 (6)	0.0369 (6)	−0.0001 (5)	0.0099 (5)	−0.0018 (5)
C3	0.0736 (10)	0.0533 (9)	0.0353 (7)	−0.0010 (7)	0.0141 (7)	0.0050 (6)
C15	0.0506 (8)	0.0447 (8)	0.0452 (7)	−0.0065 (6)	0.0059 (6)	0.0028 (6)
C6	0.0535 (8)	0.0408 (7)	0.0370 (7)	−0.0022 (6)	0.0035 (6)	0.0048 (5)
C11	0.0593 (9)	0.0468 (8)	0.0380 (7)	−0.0041 (6)	0.0106 (6)	−0.0038 (6)
C13	0.0682 (10)	0.0733 (12)	0.0418 (8)	0.0019 (8)	0.0088 (7)	0.0161 (7)
C2	0.0733 (10)	0.0446 (8)	0.0422 (7)	−0.0109 (7)	0.0144 (7)	0.0013 (6)
C12	0.0662 (10)	0.0706 (11)	0.0355 (7)	−0.0048 (8)	0.0067 (7)	−0.0033 (7)
C14	0.0665 (10)	0.0543 (9)	0.0559 (9)	−0.0069 (8)	0.0106 (8)	0.0155 (7)

### Geometric parameters (Å, °)

**Table:**

Cl1—C4	1.7406 (14)	C10—C11	1.3923 (19)
N2—C7	1.3806 (17)	C10—C15	1.394 (2)
N2—N1	1.4042 (15)	C10—C9	1.4635 (18)
N2—C1	1.4169 (16)	C3—C2	1.382 (2)
N1—C9	1.2897 (17)	C3—H3	0.9300
C7—O1	1.2126 (17)	C15—C14	1.384 (2)
C7—C8	1.504 (2)	C15—H15	0.9300
C8—C9	1.4900 (18)	C6—H6	0.9300
C8—H8A	0.9700	C11—C12	1.384 (2)
C8—H8B	0.9700	C11—H11	0.9300
C1—C6	1.3824 (19)	C13—C12	1.377 (3)
C1—C2	1.3894 (19)	C13—C14	1.382 (3)
C4—C3	1.367 (2)	C13—H13	0.9300
C4—C5	1.378 (2)	C2—H2	0.9300
C5—C6	1.383 (2)	C12—H12	0.9300
C5—H5	0.9300	C14—H14	0.9300
			
C7—N2—N1	112.65 (11)	N1—C9—C8	112.52 (11)
C7—N2—C1	128.96 (11)	C10—C9—C8	125.29 (12)
N1—N2—C1	118.37 (10)	C4—C3—C2	119.75 (14)
C9—N1—N2	107.72 (11)	C4—C3—H3	120.1
O1—C7—N2	126.65 (14)	C2—C3—H3	120.1
O1—C7—C8	128.47 (13)	C14—C15—C10	120.14 (15)
N2—C7—C8	104.88 (11)	C14—C15—H15	119.9
C9—C8—C7	102.15 (11)	C10—C15—H15	119.9
C9—C8—H8A	111.3	C1—C6—C5	119.88 (13)
C7—C8—H8A	111.3	C1—C6—H6	120.1
C9—C8—H8B	111.3	C5—C6—H6	120.1
C7—C8—H8B	111.3	C12—C11—C10	120.47 (15)
H8A—C8—H8B	109.2	C12—C11—H11	119.8
C6—C1—C2	119.68 (13)	C10—C11—H11	119.8
C6—C1—N2	119.22 (12)	C12—C13—C14	119.98 (15)
C2—C1—N2	121.10 (12)	C12—C13—H13	120.0
C3—C4—C5	120.84 (13)	C14—C13—H13	120.0
C3—C4—Cl1	120.53 (11)	C3—C2—C1	120.03 (14)
C5—C4—Cl1	118.63 (12)	C3—C2—H2	120.0
C4—C5—C6	119.79 (14)	C1—C2—H2	120.0
C4—C5—H5	120.1	C13—C12—C11	120.14 (16)
C6—C5—H5	120.1	C13—C12—H12	119.9
C11—C10—C15	118.94 (13)	C11—C12—H12	119.9
C11—C10—C9	119.80 (13)	C13—C14—C15	120.32 (16)
C15—C10—C9	121.26 (13)	C13—C14—H14	119.8
N1—C9—C10	122.18 (12)	C15—C14—H14	119.8
			
C7—N2—N1—C9	−1.81 (16)	C15—C10—C9—C8	173.39 (14)
C1—N2—N1—C9	179.69 (11)	C7—C8—C9—N1	1.93 (16)
N1—N2—C7—O1	−176.48 (15)	C7—C8—C9—C10	−179.50 (13)
C1—N2—C7—O1	1.8 (3)	C5—C4—C3—C2	−0.7 (2)
N1—N2—C7—C8	2.95 (16)	Cl1—C4—C3—C2	178.94 (13)
C1—N2—C7—C8	−178.75 (13)	C11—C10—C15—C14	−0.4 (2)
O1—C7—C8—C9	176.62 (16)	C9—C10—C15—C14	179.13 (14)
N2—C7—C8—C9	−2.79 (15)	C2—C1—C6—C5	−1.1 (2)
C7—N2—C1—C6	−160.62 (14)	N2—C1—C6—C5	179.36 (13)
N1—N2—C1—C6	17.60 (18)	C4—C5—C6—C1	−0.5 (2)
C7—N2—C1—C2	19.9 (2)	C15—C10—C11—C12	1.2 (2)
N1—N2—C1—C2	−161.90 (13)	C9—C10—C11—C12	−178.37 (13)
C3—C4—C5—C6	1.4 (2)	C4—C3—C2—C1	−1.0 (3)
Cl1—C4—C5—C6	−178.24 (12)	C6—C1—C2—C3	1.9 (2)
N2—N1—C9—C10	−178.86 (11)	N2—C1—C2—C3	−178.65 (14)
N2—N1—C9—C8	−0.24 (15)	C14—C13—C12—C11	0.6 (3)
C11—C10—C9—N1	171.35 (13)	C10—C11—C12—C13	−1.3 (3)
C15—C10—C9—N1	−8.2 (2)	C12—C13—C14—C15	0.2 (3)
C11—C10—C9—C8	−7.1 (2)	C10—C15—C14—C13	−0.3 (3)

### Hydrogen-bond geometry (Å, °)

**Table:**

Cg1 is the centroid of the C10–C15 ring.

**Table:**

*D*—H···*A*	*D*—H	H···*A*	*D*···*A*	*D*—H···*A*
C8—H8B···O1^i^	0.97	2.40	3.3115 (19)	156
C8—H8A···Cg1^ii^	0.97	2.76	3.5026 (17)	134

Symmetry codes: (i) *x*, −*y*+3/2, *z*+1/2; (ii) −*x*+2, −*y*+1, −*z*+2.

### Table 2 Comparison of C═O and N—N bond lengths (Å) between the title compound and reported pyrazolone compounds.

**Table:**

Compound	C═O	N—N
C_13_H_14_N_2_O_2_^a^	1.313 (2)	1.395 (2)
C_19_H_16_N_2_O_2_^a^	1.261 (2)	1.404 (2)
C_15_H_12_N_2_O_2_S^a^	1.246 (2)	1.373 (2)
C_22_H_15_ClN_2_O^c^	1.228 (2)	1.405 (2)
C_16_H_11_N_3_O^c^	1.252 (3)	1.412 (4)
C_16_H_10_ClN_3_O^c^	1.250 (5)	1.420 (5)
C_10_H_8_N_4_O_5_^d^	1.207 (3)	1.412 (2)
C_15_H_11_ClN_2_O^e^	1.213 (2)	1.404 (2)

Notes: (a) Holzer *et al.* (1999); (b) Bovio *et al.* (1974); (c) Ferretti *et al.* (1985); (d) Dardonville *et al.* (1998); (e) this work.
